# Monitoring oil spill in Norilsk, Russia using satellite data

**DOI:** 10.1038/s41598-021-83260-7

**Published:** 2021-02-15

**Authors:** Sankaran Rajendran, Fadhil N. Sadooni, Hamad Al-Saad Al-Kuwari, Anisimov Oleg, Himanshu Govil, Sobhi Nasir, Ponnumony Vethamony

**Affiliations:** 1 Environmental Science Center, Qatar University, P.O. Box: 2713, Doha, Qatar; 2 Department of Climate Change, State Hydrological Institute, St. Petersburg, Russia; 3 Department of Applied Geology, National Institute of Technology, Raipur, India; 4 Earth Science Research Center, Sultan Qaboos University, Al-Khod, 123 Muscat, Oman

**Keywords:** Natural hazards, Environmental monitoring, Environmental impact

## Abstract

This paper studies the oil spill, which occurred in the Norilsk and Taimyr region of Russia due to the collapse of the fuel tank at the power station on May 29, 2020. We monitored the snow, ice, water, vegetation and wetland of the region using data from the Multi-Spectral Instruments (MSI) of Sentinel-2 satellite. We analyzed the spectral band absorptions of Sentinel-2 data acquired before, during and after the incident, developed true and false-color composites (FCC), decorrelated spectral bands and used the indices, i.e. Snow Water Index (SWI), Normalized Difference Water Index (NDWI) and Normalized Difference Vegetation Index (NDVI). The results of decorrelated spectral bands 3, 8, and 11 of Sentinel-2 well confirmed the results of SWI, NDWI, NDVI, and FCC images showing the intensive snow and ice melt between May 21 and 31, 2020. We used Sentinel-2 results, field photographs, analysis of the 1980–2020 daily air temperature and precipitation data, permafrost observations and modeling to explore the hypothesis that either the long-term dynamics of the frozen ground, changing climate and environmental factors, or abnormal weather conditions may have caused or contributed to the collapse of the oil tank.

## Introduction

The oil spill detection and tracking through satellite sensors have remarkable advances in utilizing the visible, shortwave to thermal infrared (optical) and the microwave radar bands. Surface identification and mapping of an oil spill are essential to evaluate the potential spread and float from the source to the adjacent areas or endpoints^1,2^. The ability to characterize the interactions of incident oil spills is critical for detecting the spill in both the optical and radar bands. Studies have demonstrated the use of both active and passive sensors to detect, map, and monitor oil spills^3–7^. Several researchers have conducted the analyses and modeling of oil spills that occurred in different seas. For example, Marmara Sea^8^, Mediterranean Sea^9^, Italian Seas^10–12^, German North Sea^13^, Mexico Gulf^14^, South Aegean (Crete)^15^, Lebanon shoreline^16^, Arabian Sea^17^_,_ and coastlines of different parts of the world^18–21^. Several models were built to address the environmental dynamics of spills^22–25^.

Literature reviews show that the utilization of optical satellite remote sensing for oil spills has endeavored in numerous studies, and detection of the oil spills using the satellite data provides opportunities to map the large-scale oil pollution^26–29^. Several studies characterized the reflectance properties of different oils utilizing multispectral data^30,31^. The thickness of the oil spill has been estimated and reported in several studies^29,32–34^. The finest spectral resolution of hyperspectral remote sensing at the nanometer (nm) level was also utilized to identify spectral characteristics of oil spills^34–38^. Research studies showed the potential use of Sentinel-2 data to detect and map the oil spills spread over the gulf or sea^30,39,40^.

**Figure 1:**
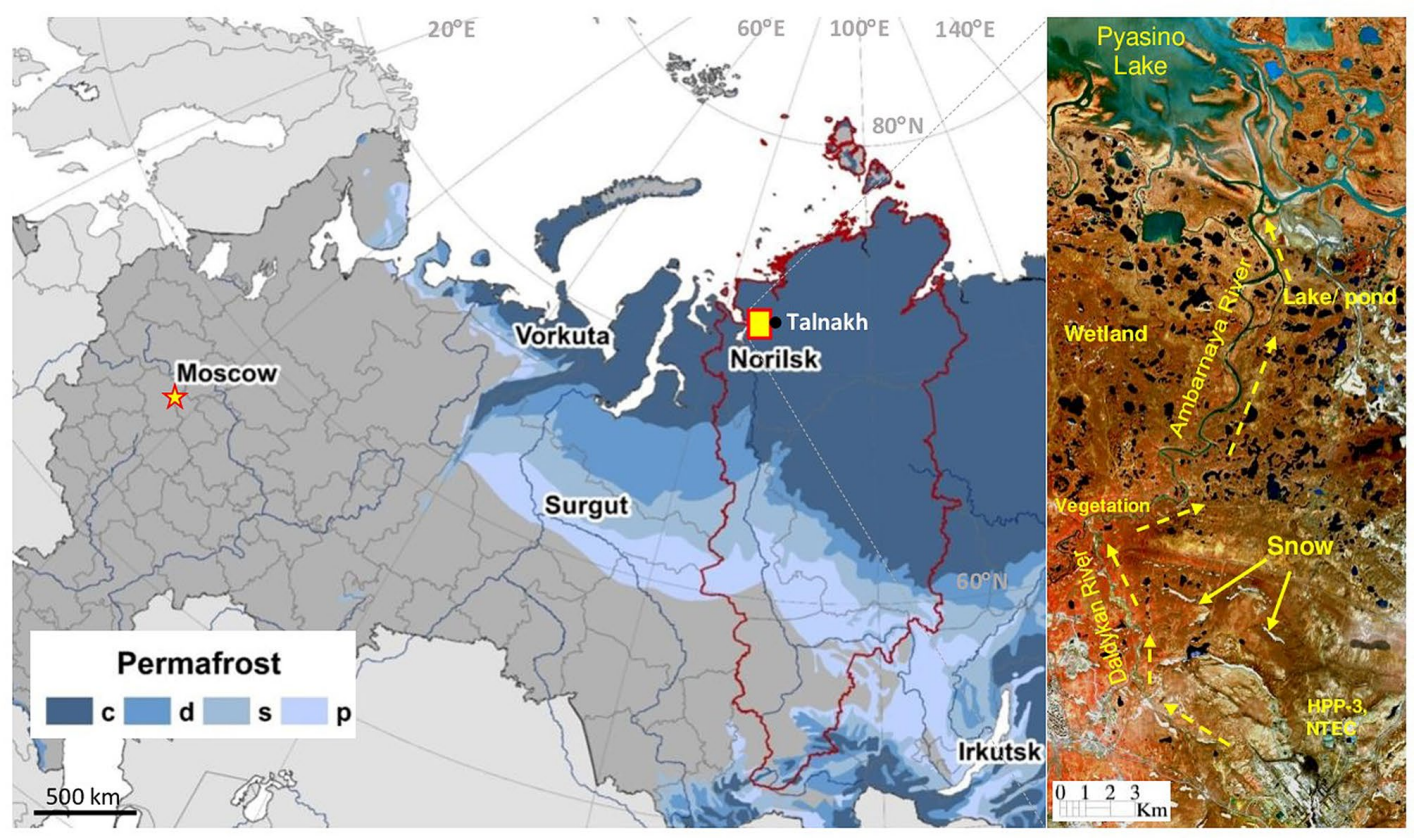
Study area around Norilsk in Krasnoyarskiy Krai, the Federal district of Russia (the border is delineated in red), and distribution of permafrost zones: c—continuous (occupies more than 90% of land), d—discontinuous (50% – 90%), s—sporadic (10% – 50%), p—isolated patches (less than 10%) [Russian Foundation for Basic Research, Project no 18-05-60005]. Insert: Sentinel-2 image [R:6; G;4; B:3, dated May 23, 2020] shows the oil spill direction from the Heat and Power Plant № 3 [HPP-3] of Norilsk-Taimyr Energy Company [NTEC] through the Daldykan River and Ambarnaya River [ENVI 5.5 https://www.harrisgeospatial.com; https://sentinel.esa.int/].

The present study explores and maps the oil spill that occurred on May 29, 2020 near the Norilsk City in the Taimyr region, Russia using Sentinel-2 data (Fig. 1). The oil spill has occurred due to the failure of a diesel fuel storage tank built upon permafrost^41,42^ and led to contamination of the water and soil over the large territories. This study has the following objectives: (1) to review the spectral absorption characteristics of the oil spill using Sentinel-2 data; (2) to map the oil spill using different image processing methods and Sentinel-2 data acquired before, during, and after the incident; (3) to distinguish the associated land surface features, especially snow, ice, water, vegetation and wetlands, and (4) to analyze the daily meteorological records from Norilsk weather station, perform permafrost modeling, and evaluate the potential roles of the climate change, thawing permafrost, and abnormal weather conditions in the occurrence of the incident.

## Spectral signatures of oil spills over the water

Several authors studied the spectra of different oil types, such as crude, diesel, lubricant, and kerosene^37,38,43,44^, and used their discernible differences to map oil spills by hyperspectral remote sensing technique^27,34–36,45,46^. Oil film has a reflectance contrast in 400–700 nm between an oil film on the sea surface and the background waters^27,47,48^.

Lu et al. measured the reflectance spectrum of oil slick using a high-resolution spectroradiometer (ASD FieldSpec Pro FR) and stated that the infrared band from 1150 to 2500 nm can be used to distinguish between the oil slick and the seawater^49^. The spectral characteristics of the oil slick are distinct at 550 and 645 nm, which are the best wavelengths for monitoring the offshore oil slick and estimating its thickness. Lu et al. studied the hyperspectral data and stated that the very thin oil slicks can change reflectance at 440 nm, which implies that the other bands to distinguish oil slicks are 350–440 nm^50^. Klemas described an oil sheen as silvery and stated that it reflects light over a wide spectral region^51^. Heavy oil appears brown, peaking at 600–700 nm in wavelength, while mousse looks red-brown and peaks closer to 700 nm. Clark et al. found that the thick oil (for example, crude oil and water in oil emulsions) in the Near Infrared (NIR) band has multiple peaks in reflectance from 1 to 1.5 µm and diagnostic organic C-H absorptions at 1.2, 1.7, and 2.3 µm, which can be used to determine oil to-water ratio and minimum oil emulsion thicknesses^52^.

Sun et al. studied Daqing crude oil, Jilin crude oil, heavy oil, and seawater from Dalian Bay by multi-angle hyperspectral polarized reflectance and found that the degree of polarization of seawater is higher than that of oil slicks in the 400–1000 nm range with little difference at 785 nm^45^. It allows distinguishing the changes of oil-slick thickness by the degree of polarization at 785 and 880 nm in the near-infrared band and thus monitoring sea-surface pollution through remote sensing. Lu et al. studied the spectra of a very thin oil slick, and found that their spectral repentances were higher than background seawater in the spectral range from 400 to 1000 nm and showed “high brightness” in visual characteristics^53^. Polychronis and Vassilia stated that the hyperspectral imagery showed oil spills brighter than seawater in all the CASI‐550 acquired bands^54^. They observed the blue‐green region of the spectrum (400–600 nm) and found that the bottom interference is minimized at 600 nm and eliminated at wave length greater than 660 nm. The spectral range 660–760 nm (upper red to near-infrared region) is the best for oil spill detection in coastal areas. Above 760 nm (near-infrared region), the water reflectance significantly drops and the image suffers from noise making it difficult to identify the oil spill. They mentioned that most of the very high-resolution multispectral sensors have bands that span across this red‐near infrared region (660–760 nm) and it should be possible to observe any oil spill occurrences. In this study we collected image spectra of the oil spill and the associated major land features such as snow and ice, water, vegetation, and wetland using the spectral bands of Sentinel-2. We explored their spectral band absorption characters and discriminated the individual features using suitable image processing methods.

## Oil spill in Norilsk region

The diesel oil spill has occurred on May 29, 2020, due to the collapse of the diesel fuel storage tank at Heat and Power Plant № 3 (HPP-3) in the Kayerkan neighborhood of the city of Norilsk^41,42,55^. The plant was operated by Norilsk-Taimyr Energy Company (NTEC) and the oil spill was an industrial disaster that released 21,000 cubic meters (17,500 tons) of diesel oil in the local Daldykan and Ambarnaya rivers^55^. The oil drifted downstreams by 12 km from the incident site and drained into the Ambarnaya River through its tributary Daldykan River. The Ambarnaya River flows into Pyasino Lake, which feeds the Pyasino River. The spill has contaminated an area of 350 km^2^ and directly affected an area of about 0.18 km^2^ near the Daldykan River^56^. Response teams have used a floating dam and booms to block the further movement of the oil over the Ambarnaya River (see “Validation of Sentinel-2 mapping” section). Clean-up efforts were complicated by the absence of roads or rivers (too shallow for boats and barges). According to the BBC News Russia^57^, the 2020 oil spill in Norilsk has been the second largest in Russian history following the spill of 94,000 tons of oil in 1994 due to the pipeline failure in the Komi Republic in North-European Russia. The cost of immediate emergency measures was about US$146 million, while the total clean-up would take five to ten years and may cost up to US$1.5 billion. While the NTEC claimed that the incident was caused by the thawing permafrost and global climate change, the Russian federal officials stated that other factors, among which is the improper operational management of the construction, played the pivotal role. The oil tank was too old; engineering survey in 2016 indicated that it required partial rebuilding, which was planned to start in June 2020^57^.

### Study area

**Figure 2:**
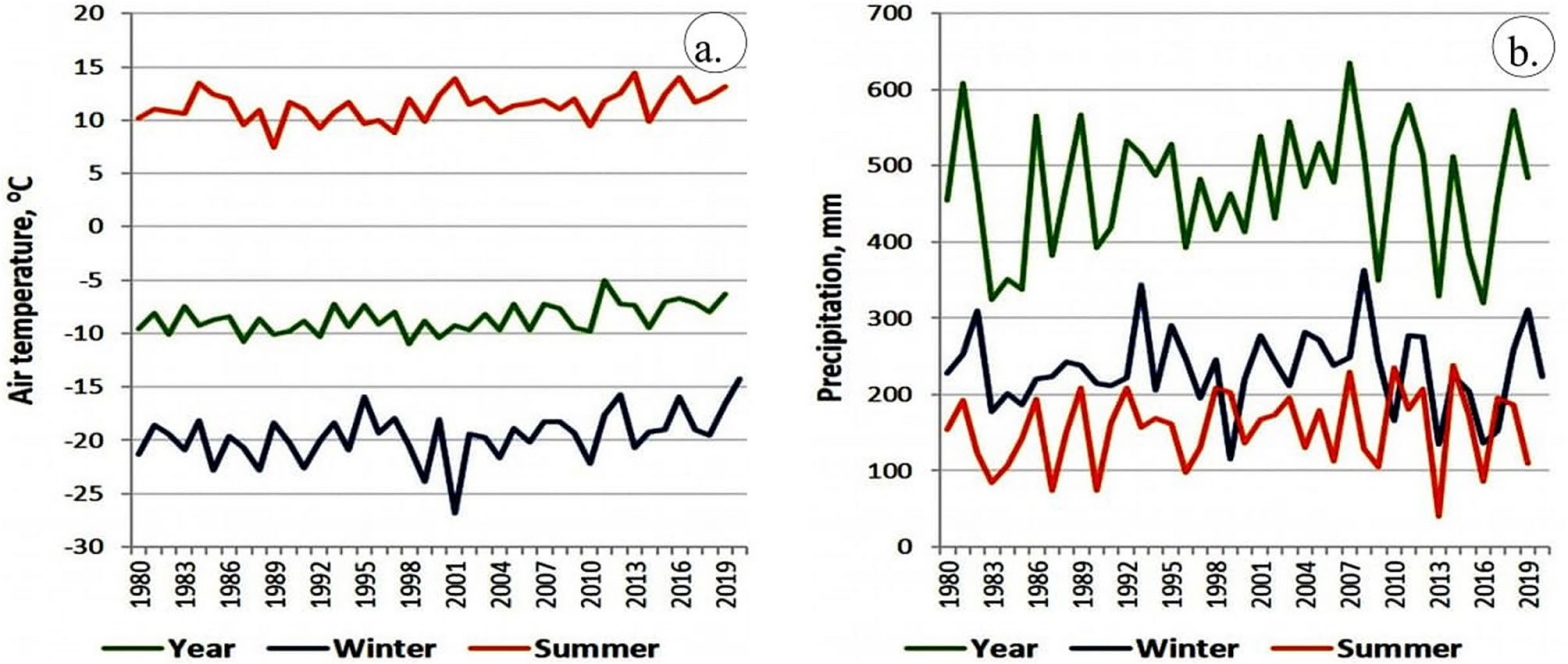
(**a**) Mean annual, winter and summer air temperature and (**b**) precipitation in Norilsk in the period 1980–2020 [Russian Foundation for Basic Research, Project no 18-05-60005].

The study area (insert in Fig. 1) is centered at the HPP-3. The area falls into the forest-tundra vegetation zone and is characterized by the severe Arctic climate with very cold winter and short summer. Graphs in Fig. 2 illustrate air temperature and precipitation records at Norilsk weather station during 1980–2020; summary statistics of the climate data is given in Table 1. These data demonstrate an accelerating warming in all seasons in the recent decades, most of which is attributed to the rising daily minimum temperatures. Statistically significant at 95% level, the minimum temperature trends range from 0.6 °C/10a in the summer to 1.05 °C/10a in winter, while the daily maximum temperature and precipitation trends are small and statistically insignificant.

**Table 1:** Statistics of the mean annual, winter and summer air temperature and precipitation sums for Norilsk [T_a_, T_min_ and T_max_ designate averaged over the season daily mean, minimum and maximum air temperatures; a – linear trend coefficient for the period 1981–2020; °C/10 years for air temperature and mm/10 years for precipitation sums’ Bold italic designate trends significant at 95% level] [Russian Foundation for Basic Research, Project no 18–05-60005].

Season	Period	T_a_, °C	T_min_, °C	T_max_, °C	P, mm
Year	1981–2020	− 8.6	− 12.5	− 4.4	469.6
	2011–2020	− 7.2	− 10.7	− 3.3	461.5
	a	***0.53***	***0.82***	0.15	6.15
Winter	1981–2020	− 19.5	− 23.7	− 15.2	232.9
	2011–2020	− 17.7	− 21.4	− 14.0	220.0
	a	***0.69***	***1.05***	0.27	− 1.43
Summer	1981–2020	11.4	7.4	15.8	155.3
	2011–2020	12.4	8.7	16.7	157.4
	a	***0.46***	***0.60***	0.22	6.17

The region is located in the continuous permafrost zone, where frozen ground occupies more than 90% of the area. Below the depth of seasonal thawing, typically less than 1 m, ground has negative temperature all year round, except under the large water bodies, such as lakes and rivers, which do not freeze up to the bottom. Such unfrozen vertical “tunnels” are called taliks, they play important role in providing pathways for the transport of water, other liquid substances and gases between the surface and deeper soil layers^58^. The study area has many small (less than 1.0 km^2^) and shallow (2–5 m) thermokarst lakes and ponds in local ground depressions with taliks underneath them. The tributary of the Ambarnaya River, the Daldykan River, flows northward into the Pyasino Lake. The riverbed consists of glacial tills, fluvial gravels and pebbles^59^. The Quaternary deposits are cemented by ice and clayey soil and peat. The presence of sandy, sandy loam, and gravel deposits are rare. The clayey soil constitutes 67% of the total area in the Norilsk region. The ice content of the frozen clayey soil varies in the range 15–45%, and the bearing capacity of the seasonally thawed soil is small, which complicates construction of the engineered structures.

Most structures in permafrost terrain are based on pile foundations. Metal piles of different lengths, typically between 6 and 12 m, are embedded into permafrost and anchored in soil by cementing ground ice. Adfreeze bond strength and bearing capacity of any given pile depend on permafrost temperature and decrease with warming of the frozen ground. Several studies demonstrated that the bearing capacity of the pile foundations in the Russian Arctic cities has already reduced in the past 50 years due to climate change damaging hundreds of engineered structures, such as buildings, pipelines, railroads, airport runways, and technical facilities^60–67^. Due to climate warming, the bearing capacity of the pile foundations in Norilsk constructed in 1970s was reduced by 10% – 15% by 2010^61^, approaching the safety threshold, typically 20%, incorporated into the initial design.

## Satellite data and image processing methods

### Sentinel-2 data

**Figure 3:**
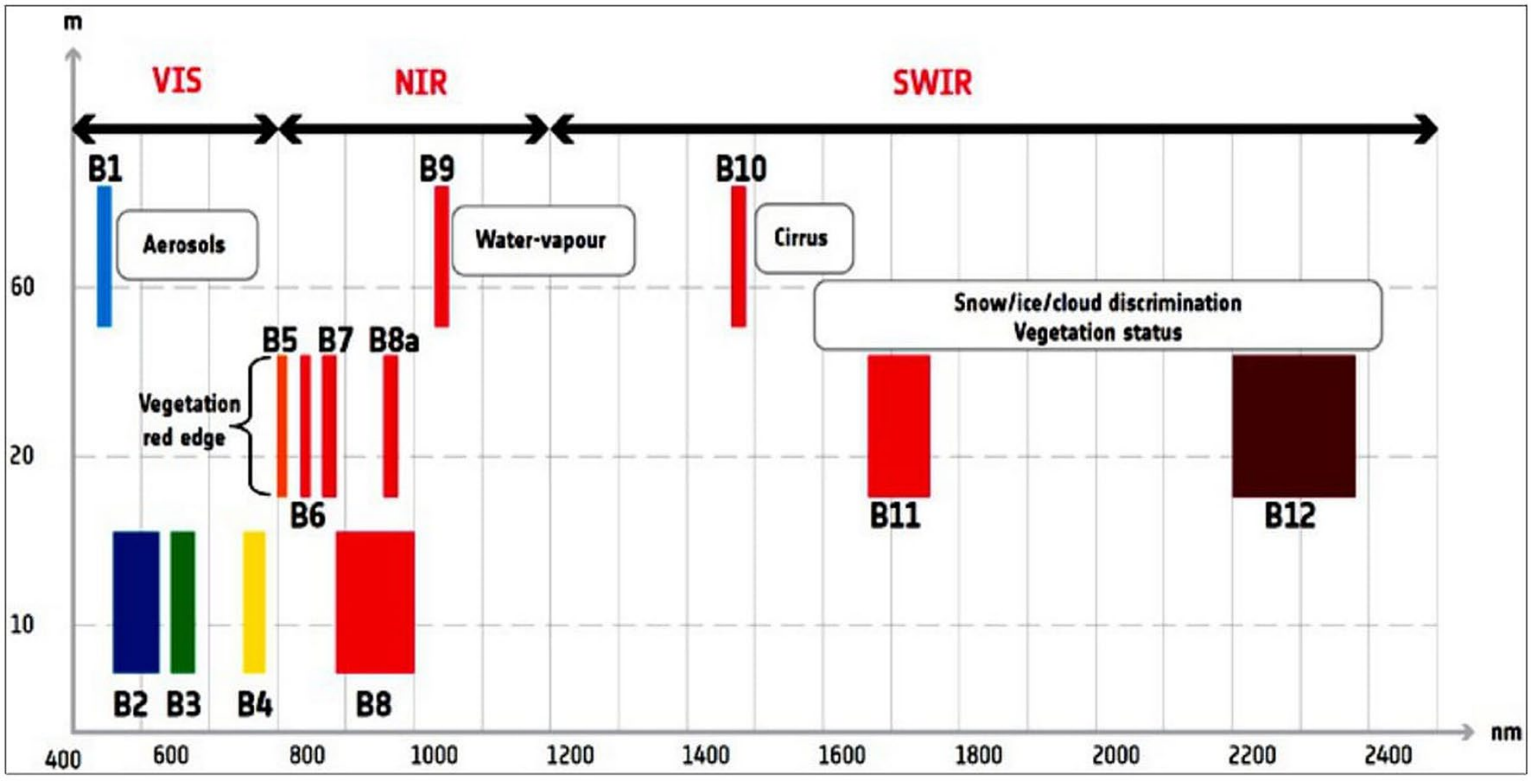
Sentinel-2 MSI spectral bands vs. spatial resolution with corresponding Full Width at Half Maximum [FWHM]^70^ [ENVI 5.5 https://www.harrisgeospatial.com; https://sentinel.esa.int/].

This study uses the data of Multi-Spectral Instruments (MSI) of Sentinel-2 available since June 2015. Sentinel-2A was launched on June 23, 2015 and Sentinel-2B on March 7, 2017 into circular sun-synchronous 786 km orbits with 98.62° inclination. They have equatorial crossing times of 10:30 a.m. with a phase delay of 180°^68^. The data are acquired with a 20.6° field of view for providing an approximately 290 km swath, and the equatorial repeat cycle of each Sentinel-2 sensor is about ten days, and five days when combined^69^. MSI has high spatial (10 m, 20 m, and 60 m) and 12-bit radiometric resolutions. The sensor senses energy that comes from the earth surface in the spectral bands 1 to 9 (including band 8a) in the visible near-infrared (VNIR) wavelength (0.44 to 0.95 µm) and spectral bands 10 to 12 in the shortwave infrared (SWIR) wavelength (1.57 to 2.28 µm). Among the bands, the three bands 1 (443 nm), 9 (945 nm), and 10 (1375 nm) have 60 m spatial resolution and dedicated for aerosol correction, water vapor, and cirrus clouds detection, respectively. The four bands 2 (Blue, 490 nm), 3 (Green, 560 nm), 4 (Red, 665 nm), and 8 (Near Infra-Red, 842 nm) have 10 m spatial resolution and meet the basic requirements for land classification. Another four bands 5 (705 nm), 6 (740 nm), 7 (783 nm), and 8a (865 nm) have 20 m spatial resolution and are dedicated to vegetation detection. The bands 11 (1610 nm) and 12 (2190 nm) in the SWIR are useful for snow/ice/cloud detection (Fig. 3; Table 2)^70,71^. Sentinel-2 data are freely downloadable from the Sentinel Hub developed by the European Space Agency (ESA) (https://sentinel.esa.int/web/sentinel/sentinel-data-access). ESA provides satellite information analysis software ‘Sentinel Application Platform (SNAP)’ which reads all the information of the Sentinel series and provides and exports data to other relative analyses comparable with Quantum GIS (QGIS 3.14, https://qgis.org/) and Environment for Visualizing Images (ENVI 5.5, Harris Geospatial Solutions, Broomfield, CO, USA; https:// www.harrisgeospatial.com) software’s.

**Table 2:** Sensor characters of Sentinel-2 bands according to the Copernicus derived user requirements^71^.

Bands	Wavelength regions	Center Wavelength (nm)	Band width (nm)	Spatial Resolution (m)	Purpose
1	VNIR	443	20	60	Atm. correction (aerosol scattering)
2		490	65	10	Vegetation senescing, carotenoid, browning and soil background; atm. correction (aerosol scattering)
3		560	35	10	Green peak, sensitive to total chlorophyll in vegetation
4		665	30	10	Max. chlorophyll absorption
5		705	15	20	Red edge position; consolidation of atmospheric corrections/fluorescence baseline
6		740	15	20	Red edge position; atmospheric correction; retrieval of aerosol load
7		783	20	20	LAI^a^; edge of the NIR plateau
8		842	115	10	LAI
8a		865	20	20	NIR plateau, sensitive to total chlorophyll, biomass, LAI, and protein; water vapor absorption reference; retrieval of aerosol load and type
9		945	20	60	Atm. correction (water vapour absorption)
10	SWIR	1375	30	60	Atm. correction (detection of thin cirrus)
11		1610	90	20	Sensitive to lignin, starch and forest above-ground biomass; snow/ice/cloud separation
12		2190	180	20	Assessment of Mediterranean vegetation conditions; distinction of clay soils for monitoring of soil erosion; distinction between live biomass, dead biomass, and soil, e.g. for burn scars mapping

^a^LAI = Leaf Area Index.

Sentinel-2 supports the continuity of the SPOT and Landsat missions and provides operational products for land-cover maps, land-cover change detection maps, and geochemical/physical variables^69^. Especially, the four bands at 10 m resolution ensure the compatibility with SPOT 4 and 5 and meet the users’ requirements for land cover classification. Numerous studies demonstrated implications of Sentinel-2 data in agriculture^72,73^, vegetation^74^, geology^71,75^, water depth retrieval^76^, land use and land cover changes^77,78^. Our study utilizes the available Sentinel-2 data at ESA portal for the cloud-free conditions in the study area acquired before, during, and after the oil spill from May 18 to July 10, 2020 (Table 3). The data were pre-processed and georeferenced to the WGS-84 ellipsoid and Universal Transverse Mercator (UTM) Zone 45 N projection and layers were staked using the QGIS 3.14 (https://qgis.org/). Subsequently, the data were subset to the area of interest and processed for mapping the oil spill occurrence using ENVI 5.5 (https:// www.harrisgeospatial.com) software.

**Table 3:** Sentinel-2 imagery used for oil mapping [https://sentinel.esa.int/].

Sensor	Date of acquisition	Image ID	Cloud cover
Sentinel-2 MSI	10072020	S2B_MSIL1C_20200710T060639_N0209_R134_T45WWT_20200710T080325	1.2035
	08072020	S2A_MSIL1C_20200708T061631_N0209_R034_T45WWT_20200708T073904	21.7875
	03072020	S2B_MSIL1C_20200703T061629_N0209_R034_T45WWT_20200703T082117	5.5783
	28062020	S2A_MSIL1C_20200628T061641_N0209_R034_T45WWT_20200628T081710	16.5355
	20062020	S2B_MSIL1C_20200620T060639_N0209_R134_T45WWT_20200620T080445	68.1177
	15062020	S2A_MSIL1C_20200615T060641_N0209_R134_T45WWT_20200615T080720	48.7731
	13062020	S2B_MSIL1C_20200613T061629_N0209_R034_T45WWT_20200613T082023	0.0
	08062020	S2A_MSIL1C_20200608T061641_N0209_R034_T45WWT_20200608T073745	0.0
	07062020	S2B_MSIL1C_20200607T055639_N0209_R091_T45WWT_20200607T075344	52.4281
	01062020	S2A_MSIL1C_20200601T062631_N0209_R077_T45WWT_20200601T074739	54.672
	31052020	S2B_MSIL1C_20200531T060639_N0209_R134_T45WWT_20200531T080653	11.3315
	23052020	S2A_MSIL1C_20200523T055641_N0209_R091_T45WWT_20200523T075418	25.8138
	21052020	S2B_MSIL1C_20200521T060629_N0209_R134_T45WWT_20200521T080553	2.4574
	18052020	S2B_MSIL1C_20200518T055639_N0209_R091_T45WWT_20200518T075132	17.8259

### Image processing

Visual imaging has been improved by the use of advanced image processing systems^79^. Green and red light bands of remotely sensed data were used as optimal bands for detecting the oil spills of sea surface^3,25,49,70,80^. Different algorithms were used to correct aberrations, color, and light levels and to process images^39,40^. In this study, the occurrence of the oil spill is studied by developing a true color composite and decorrelated image, and the occurrence of snow and ice, water, vegetation, and wetlands are studied and monitored by developing false-color composites and band ratios indices images^3,49,54,81^. The visual interpretation of images has been considered as one of the most effective methods to study the occurrence of the oil spill and associated features.

In this study true-color images are developed to detect the oil spill using the Sentinel-2 spectral bands red (B04), green (B03), and blue (B02) in the corresponding red, green and blue channels. In brief, true-color image is a good representation of the visual features as our eyes see the world, i.e. we see healthy vegetation in green.

Developing false-color composites (FCCs) using spectral bands of VNIR and SWIR regions have the potential to show image features from a combination of three spectral bands. The method develops images using three primary colors namely red, green, and blue (RGB) at a time as a color composite. The resulted image exhibits each pixel in a color determined by the combination of the RGB in different brightness and depicts features in different colors that differ from those of a true-color image. A standard FCC (examples; 8, 4, 3 of Sentinel Bands; 5, 4, 3 bands of OLI of Landsat 8; 3, 2, 1 of ASTER) shows denser plant-covered land in deep red, water in blue or black, and cities in gray. The images are commonly used to assess vegetation and plant density and health since they reflect near-infrared and green light while absorbing red. The band combinations of Sentinel-2 bands for different applications are found in the Sentinel Application Platform (SNAP) program.

Construction of band ratios and indices enhance spectral differences between bands and reduce the effects of topography. Several indices have been constructed and available at the Index database (IDB) specifically for Sentinel-2 satellite (https://custom-scripts.sentinel-hub.com/custom-scripts/sentinel-2/indexdb/). In this study, the indices namely, the Snow Water Index (SWI)^82^, Normalized Difference Water Index (NDWI)^83^, and Normalized Difference Vegetation Index (NDVI)^84–86^, were used for detecting the snow, water, vegetation and wetland and for evaluating their changes between May 18 to July 10, 2020.

*Snow Water Index (SWI):* Snow is highly reflective in the visible parts of the electromagnetic spectrum and highly absorptive in the NIR or the short-wave infrared part of the spectrum. This study uses the new Snow Water Index (SWI) proposed by Dixit et al. (2019)^82^ to study the occurrence and distribution of snow and ice in the study area. The index uses the combination of green, NIR, and SWIR bands and analyzes the spectral characteristics of snow and eliminates the impacts of cloud, soil, and vegetation. In the SWI, the water feature shows higher reflectance in the green wavelength and absorbs the maximum radiation in the NIR. The absorptive property of water in the NIR makes it possible to easily discriminate snow from water^82,87,88^. In this study we used the SWI to create a significant contrast between snow and other features, especially in the case of water^82^. Moreover, the SWI uses the snow and vegetation reflectance characters in the green and NIR wavelength to map the snow under vegetation. The snow water index (SWI) is expressed by

$$ {\text{SWI}} = \frac{{{\text{Green}}\left( {{\text{NIR}} - {\text{SWIR}}} \right){ }}}{{\left( {{\text{Green}} + {\text{NIR}}} \right)\left( {{\text{NIR}} + {\text{SWIR}}} \right)}} $$

Here, the ratio of (green/green + NIR) is used for reducing the impact of vegetation on snow and the ratio (NIR − SWIR/NIR + SWIR) works as a water mask.

*Normalized Difference Water Index (NDWI):* To understand the occurrence and distribution of water in the study area, this study uses the NDWI. The NDWI uses the green (band 3) and NIR (band 8) bands. The NDWI index is expressed by

$$ {\text{NDWI}} = \frac{{{\text{Green}} - {\text{NIR}}}}{{{\text{Green}} + {\text{NIR}}}} $$

*Normalized Difference Vegetation Index (NDVI):* Occurrence and distribution of vegetation in the study area are studied using NDVI^89^. It normalizes the ratio between red (R, band4) and near-infrared (NIR, band 8) bands^84,90^. The NDVI is used to monitor vegetation health, hydrologic stress, and the amount of biomass. Strong and well-nourished vegetation will absorb most of the received visible wavelengths and will reflect a large proportion of the NIR light, whereas poor or thin vegetation will reflect more visible wavelength light and less near-infrared light.

$$ {\text{NDVI}} = \frac{{{\text{NIR}} - {\text{Red}}}}{{{\text{NIR}} + {\text{Red}}}} $$

The occurrence and distribution of wetlands are studied using the NDVI and NDWI, and by developing FCC images using spectral bands 3, 4, and 8 of Sentinel-2^85,86^.

In this study, we also used the decorrelation stretching method^91,92^ to study the land and water resources and assess the impacts of the oil spill. The method is used to remove the high correlation commonly found in multispectral data sets. It produces a more colorful composite image by transforming the highly correlated data sets usually from a three bands input. The transformed channels can themselves be contrast stretched and arbitrarily assigned primary colors to display a color composite image^91–93^. The method has the potential to distinguish the spectral reflectance of different features of the earth surface and was utilized to map rock types using enhanced Landsat Thematic Mapper data^92,94^ and ASTER data^95^. In this study, the Sentinel-2 spectral bands 3, 8, and 11 were decorrelated to optimize the display of snow and ice, water, vegetation, and wetland resources.

The outcome of the Sentinel-2 results could be validated with post-event field photographs available in the ‘News and Press-Release’ of the Norilsk Nickel (Nornickel) Company (https://www.nornickel.com/)^41^.

## Results and Discussion

**Figure 4:**
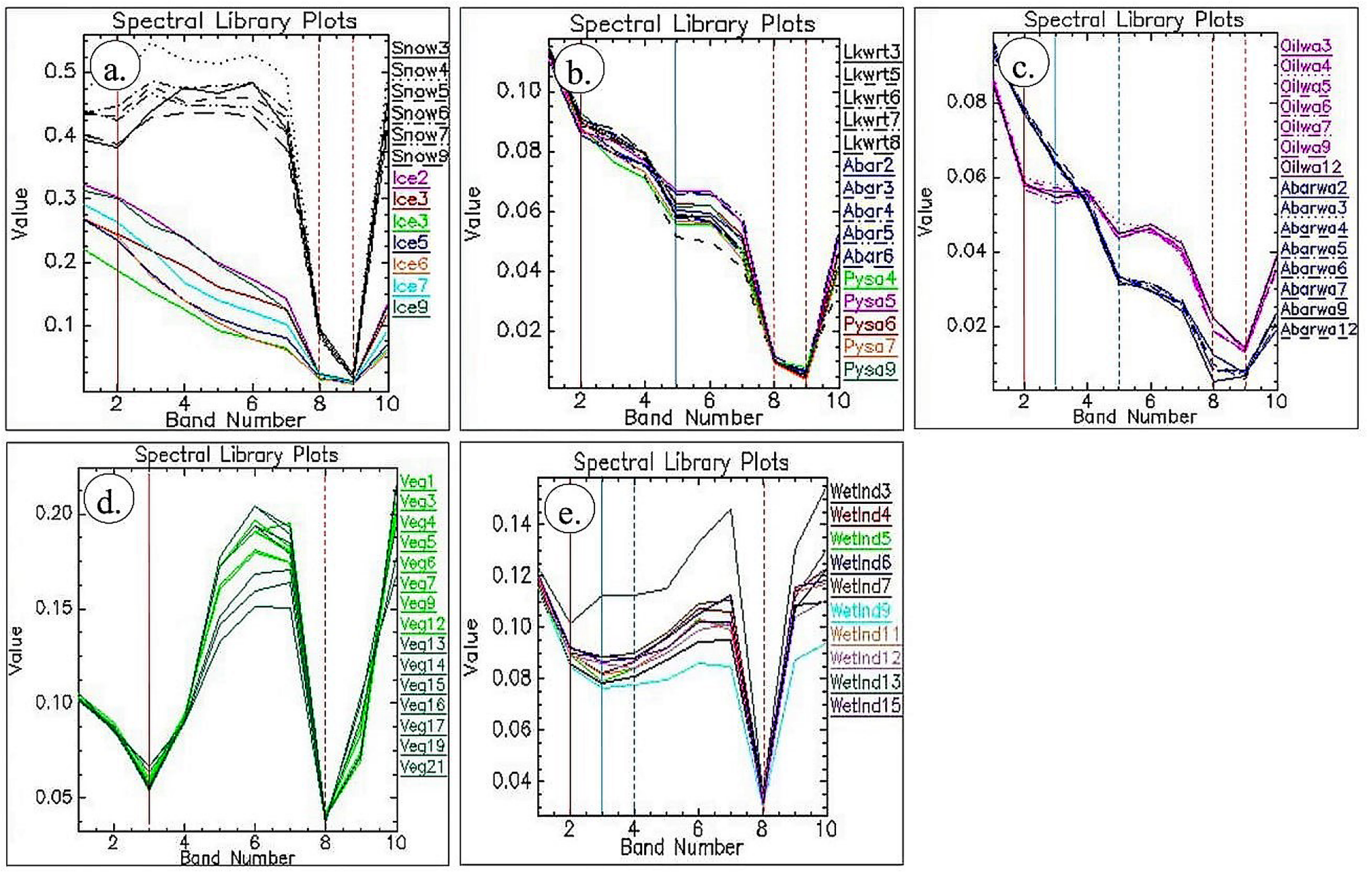
Sentinel-2 image spectra of (**a**) snow and ice, (**b**) water, (**c**) oil spill, (**d**) vegetation, and (**e**) wetland of the study area [ENVI 5.5 https://www.harrisgeospatial.com; https://sentinel.esa.int/].

The spectra of the features collected from the images are given in Fig. 4. The spectra of snow and ice pixels (Fig. 4a, black and colored, respectively) were collected over their exposures found on the land and in lakes/ponds, the Ambarnaya River, and the Pysaina Lake using the Sentinel-2 data acquired on May 18, 21 and 23, 2020. The plot shows that the spectra of snow are more reflective than ice in the visible bands and highly absorptive in the NIR and SWIR bands (Fig. 4a). The snow has spectral bands absorptions in the bands 3 (560 nm, red vertical line), 8a (865 nm, red dash-dot vertical line), and 11 (1610 nm, red dash vertical line) whereas, the ice exhibits absorptions in the bands 8a and 11. The bands could be best used to map the snow and ice of the study area.

The spectra of water collected over the lakes/ponds, the Ambarnaya River, and the Pysaino Lake using the Sentinel-2 spectral bands acquired from May 31 to July 10, 2020, are given in Fig. 4b. The plot shows that water has strong absorption in the green and near-infrared (NIR) bands and low reflection in the range from visible to infrared wavelengths when compared to the spectra of snow and ice (compare reflectance values in Fig. 4a, b). The water showed absorptions in the bands 3 (red vertical line), 6 (740 nm, blue vertical line), 8a (red dash-dot vertical line), and 11 (red dash vertical line). The spectra collected over the oil spill and water of the Ambarnaya River using the Sentinel-2 spectral bands acquired during May 31, 2020, and June 01, 2020, are given in Fig. 4c. The spectra of oil spill showed strong absorptions in the green, NIR and SWIR bands and low reflection in the range from visible to infrared wavelengths when compared to the spectra of snow and ice and water (Fig. 4a, b). The spectra of the oil spill of the river showed strong absorptions in the spectral bands 3 (red vertical line), 4 (665 nm, blue vertical line), 6 (blue dash vertical line), 8a (red dash-dot vertical line) and 11 (red dash vertical line). The spectra of the river water collected during the dates showed poor absorption in the spectral band 3 (red vertical line) and strong absorptions in the bands 6 (blue dash vertical line), 8a (red dash-dot vertical line) and 11 (red dash vertical line). The water with oil spill showed strong absorptions in the bands 3 and 4 when compared to the river water, which showed more reflection and poor absorption in the bands 3 and 4. The oil spill exhibited more reflective in the NIR and SWIR bands when compared to the river water^49,54^. The study of spectra well distinguished the spectral band absorptions characteristics of the oil spill and water of the Ambarnaya River and the bands can be used to discriminate the oil spill.

The spectra collected over vegetation of the study area using the Sentinel-2 spectral bands acquired from June 08 to July 10, 2020 are given in Fig. 4d. The plot shows that the spectra of vegetation have strong absorptions in red and NIR bands. The vegetation showed absorptions in the bands 4 (red vertical line) and 8a (red dash-dot vertical line). The spectra collected over the wetland of the study area are given in Fig. 4e. The plot shows that the spectra of wetlands have strong absorptions in green, red, and NIR bands. The wetland showed absorptions in the bands 3 (red vertical line), 4 (blue vertical line), 5 (704 nm, blue dash vertical line), and 8a (red dash vertical line). Thus, the spectral bands 3, 4, 5, 6, 8a, and 11 of Sentinel-2 can be well utilized to discriminate the oil spill and the associated major features of the study area.

### Sentinel-2 mapping of oil spill

**Figure 5:**
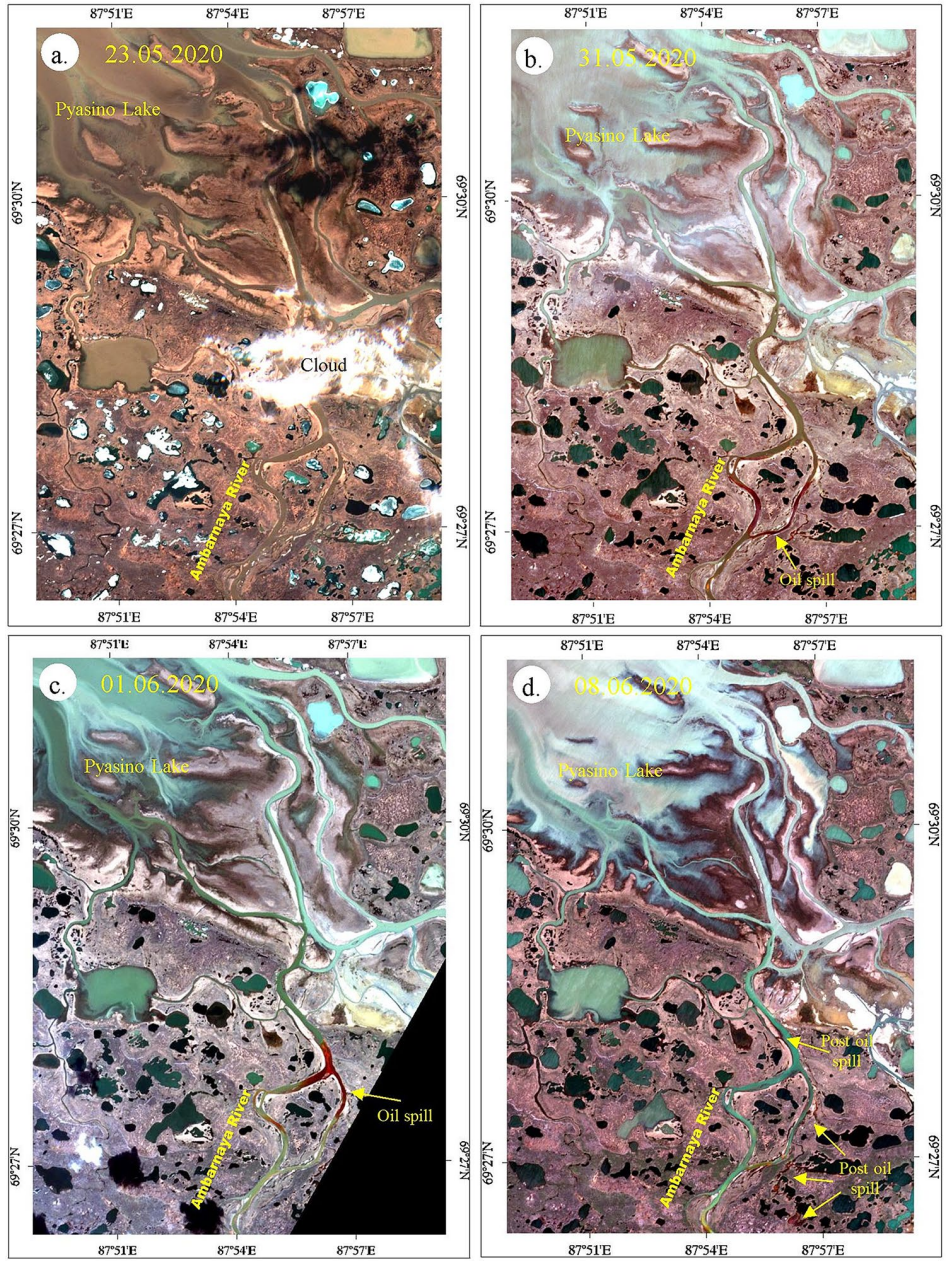
True color images [R:4; G:3; B:2] of Sentinel-2 (**a**) acquired on May 23, 2020 show the absence of oil spill in the Ambarnaya River and (**b**) and (**c**) acquired on May 31, 2020 and June 01, 2020 show oil spill in the river channel in crimson red, and (**d**) the image acquired on June 08, 2020 show the spilled oil in the creeks and water bodies found parallel to the river channel [ENVI 5.5 https://www.harrisgeospatial.com; https://sentinel.esa.int/].

o confirm the incident of the Norilsk diesel oil spill, show the spatial distribution of the spill and assess the impacts of the spillover the land, true color composites (R4; G:3; B:2) were developed and studied using Sentinel-2 data acquired before, during and after the incident (May 18 to July 10, 2020). Here, the bands 3 and 4 were chosen since the upper red to near-infrared spectral (between 660 and 760 nm) are the best for oil spill identification. The true color images of May 31 and June 01, 2020, acquired during the oil spill and the images of May 23 and June 08, 2020, acquired before and after the oil spill is given in Fig. 5. The true color images of May 31 and June 01, 2020, the spectral bands red (B4), green (B3) and blue (B2) projected in the corresponding red, green and blue channels, show the oil spill in crimson red with fine texture over the channel of Ambarnaya River^28^. The movement of oil is interpreted from their position over the images between May 31 and June 01, 2020^55^. The movement of oil from the Norilsk-Taimyr Energy Thermal and Power Plant is not studied since the images acquired before May 31, 2020, were affected by clouds over the area. The image acquired on May 23, 2020 shows the absence of the oil spill in the river channel. The image acquired on June 08, 2020 shows the presence of post-oil spill in the creeks and water bodies found parallel to the river channel. The causes of the spill are interpreted on both sides of the bank of the river channel in the shades of dark reddish-brown.

### Sentinel-2 mapping of snow and ice

**Figure 6:**
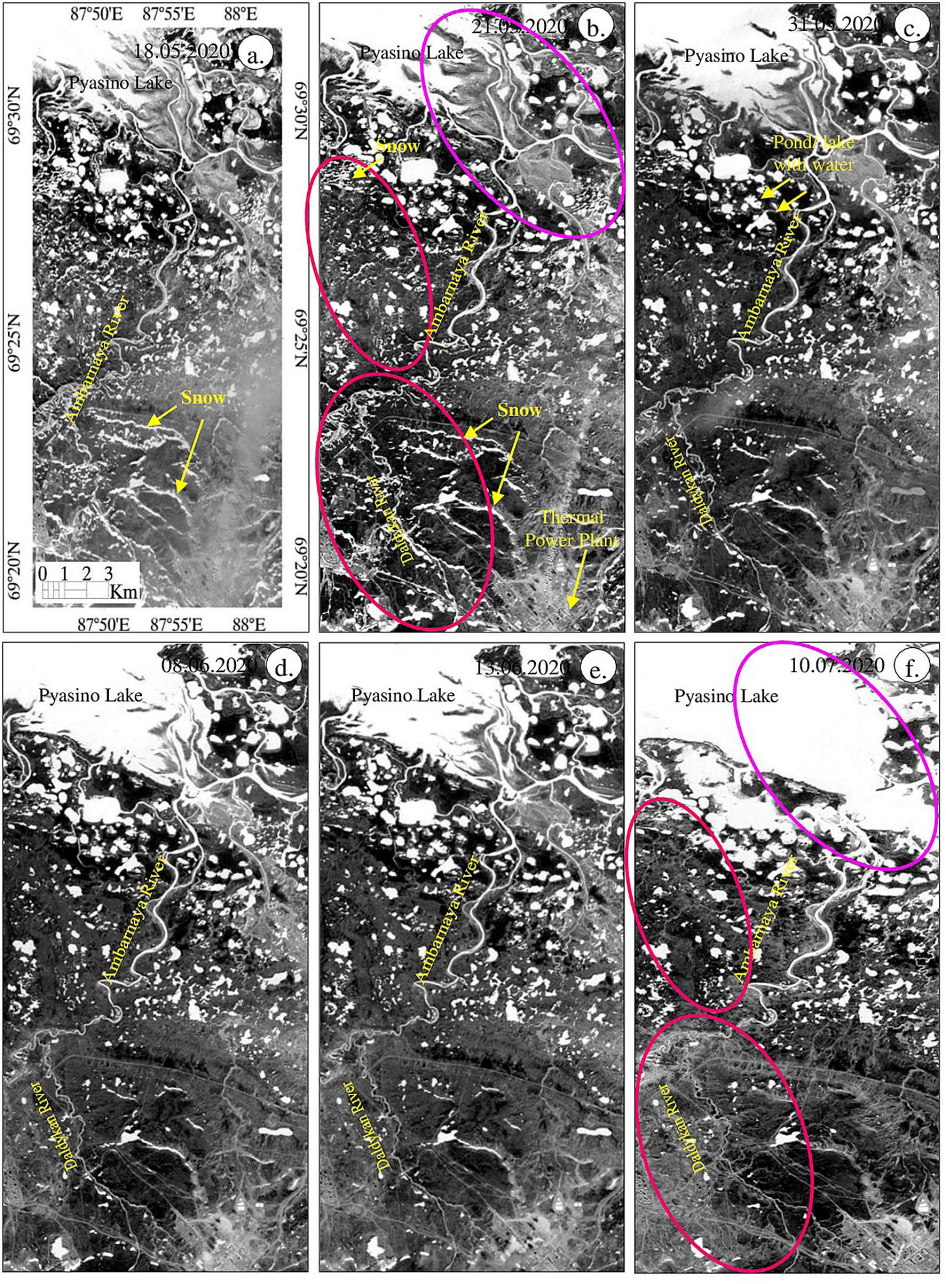
(**a**–**f**) Sentinel-2 SWI images of May 18 to July 10, 2020 [ENVI 5.5 https://www.harrisgeospatial.com; https://sentinel.esa.int/] showing the existence of snow and ice, and water resources of the study area in bright pixels.

The occurrence and distribution of snow and ice of the study area were studied using Snow Water Index (SWI). The results of SWI obtained from May 18 to July 10, 2020, are given in Fig. 6. The images show the presence of snow and ice in bright pixels and appear as white warped patches and dots with fine texture in parts of the land and near the Pyasino Lake in the images acquired on May 18 and 21, 2020 and very few or none in the images acquired on the subsequent dates. For example, the occurrence of snow and ice in the image of May 21, 2020 (see red ellipticals) can be compared and interpreted with the images of subsequent dates. The water features of the land exhibit as white spots on the land, and the Pyasino Lake appears as massive large white patches in all the images. The image on May 18, 2020 shows the area of Pyasino Lake more exposed (see magenta Ellipticals) and the gradual increase of water in the area in the subsequent dates due to the melting of snow and ice and the movement of water from the upstream. This is consistent with the daily air temperature data (see Fig. 10b in below section), which demonstrates that May in 2020 was markedly warmer than the norm, with up to 15 °C temperatures at the daytime, while normally it is below 5 °C. Unusual atmospheric warmth and large quantities of incoming solar radiation due to long daytime at high latitudes favored rapid snow and ice melt, as seen at the images. Water streams may have drained over the surface surrounding the foundation of the oil tank, leading to local ground temperature rise and erosion. Taken together, these factors may have caused the failure of the construction.

**Figure 7:**
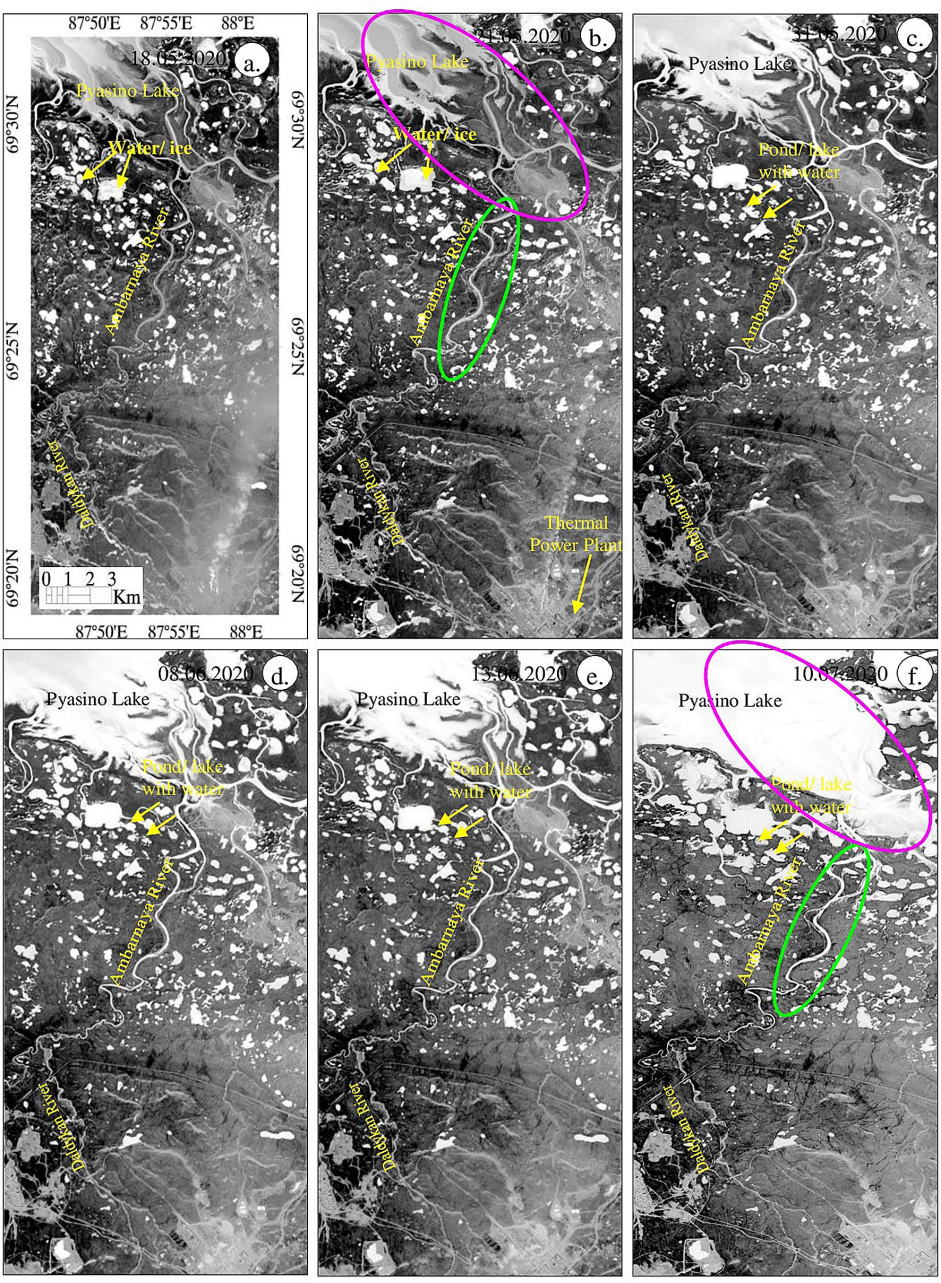
(**a**–**f**) Sentinel-2 NDWI images of May 18 to July 10, 2020 [ENVI 5.5 https://www.harrisgeospatial.com; https://sentinel.esa.int/] showing the presence of water resources as bright pixels.

We used the Normalized Difference Water Index (NDWI) to understand the occurrence and distribution of water against the background of rapid snow and ice melt. NDWI over the period from May 18 to July 10, 2020 is illustrated in Fig. 7, water appears on these images as bright pixels. Images taken after May 31, look bright due to the melting of the snow and ice in the entire watershed. Interestingly enough, the Pyasino Lake and Ambarnaya River on the images of 18 and 21 May 2020, exhibit less water and appear as light grey suggesting that they were covered by snow or ice when compared with that of the images of subsequent dates (see magenta and green ellipticals). The results of NDWI support the detection of water using SWI.

### Sentinel-2 mapping of vegetation and wetland

**Figure 8:**
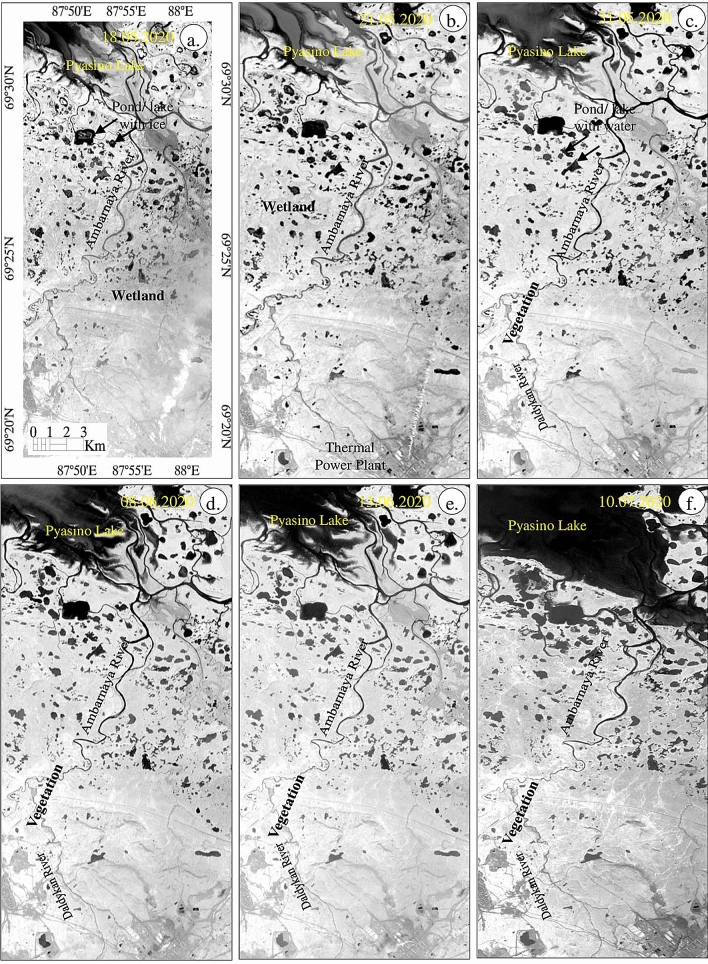
(**a**–**f**) Sentinel-2 NDVI images of May 18 to July 10, 2020 [ENVI 5.5 https://www.harrisgeospatial.com; https://sentinel.esa.int/] showing the presence of vegetation in bright pixels.

**Figure 9:**
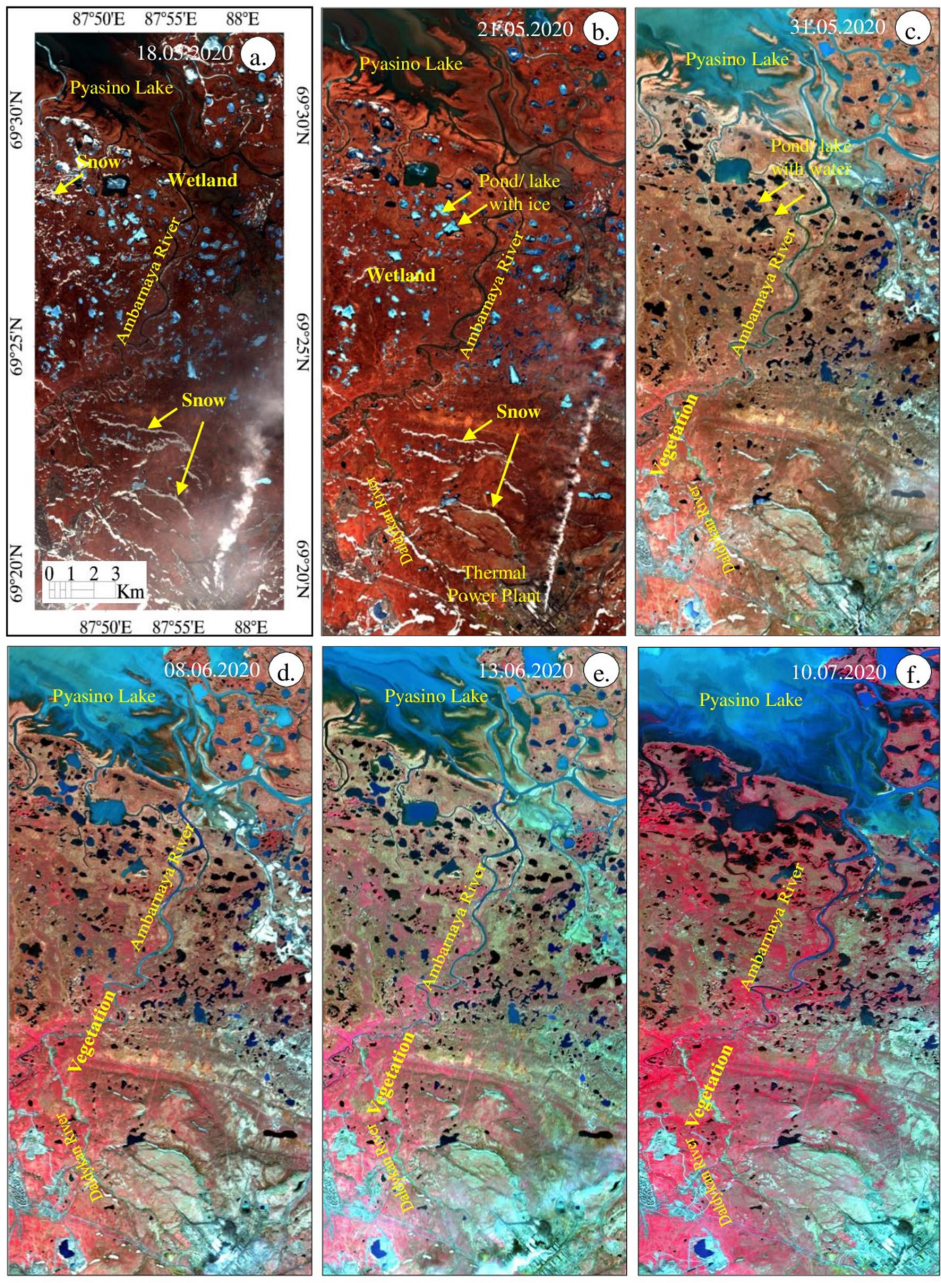
(**a**–**f**) Sentinel-2 false-color composite images (R:8; G:4; B:3) of May 18 to July 10, 2020 [ENVI 5.5 https://www.harrisgeospatial.com; https://sentinel.esa.int/] showing the presence of snow, water, vegetation, and wetland features.

Occurrence and distribution of vegetation in the study area were studied using the Normalized Difference Vegetation Index (NDVI). The results of NDVI obtained from May 18 to July 10, 2020, are given in Fig. 8. The images show the presence of vegetation in white pixels (high values) with a smooth texture and water in black pixels (low or negative values) with smooth texture from May 21 to July 10, 2020. The gradual increases of vegetation (white pixels) around the Daldykan River and along the Ambarnaya River are observed which may be due to pronounced warmth in the area before and during those dates (Fig. 2). The image of July 10, 2020 showed well established vegetation around the Pyasino Lake and water bodies, unlike the image of May 18, where these features are not pronounced. The image exhibits land in grey; changes in the tone are observed over the images that were acquired thereafter. The images of NDWI do not show the changes that interpreted over the images of NDVI. The changes represent that the land was wetter during May 18 and 21, 2020, and the wetness got decreased with the increase of vegetation thereafter due to the increase of temperature. The fumes of Thermal and Power Plant appear in a white and vertical linear pattern and can be distinguished in this image. The growth and increase of vegetation and changes in the wetness of land of the study area are further confirmed by developing FCC images using the spectral bands 8A, 4, and 3 (Fig. 9).

The FCC images (R:8; G:4; B:3) of May 18 to July 10, 2020 depict almost all the information studied from SWI, NDWI, and NDVI images. The images (Fig. 9) show the occurrence of snow as white warped patches and dots in parts of the land and near the Pyasino Lake (compare red elliptical in Fig. 6) in the images acquired on May 18 and 21, 2020. The snows have not appeared on the images acquired between May 31 and July 10, 2020. The water of the land appeared in light blue till May 21, 2020, and exhibited dark blue thereafter. The detailed interpretations show that the water features of the land have occurred as ice with water initially (until 31 May 2020, see SWI image) and later the features appeared with water due to the fact of ice melting. Additionally, the water of Pyasino Lake exhibited dark blue, blue, and light blue from May 18 to July 10, 2020 representing changes in the state of water due to an increase in temperature. The gradual increase of water in Pyasino Lake is well seen between May 31 and July 10, 2020. The increase of water in the lake is due to the melting of ice and the movement of water from the upstream of the watershed. The images show vegetation in red to dark pink with fine to medium texture on the images acquired from May 31 to July 10, 2020, and no or very poor vegetation is observed on the image of June 18 and 21, 2020. The increase of vegetation is interpreted along the Daldykan and Ambarnaya rivers and around the Pyasino Lake thereafter as interpreted on the NDVI images (Fig. 8). The increase of vegetation may be due to an increase in the temperature and water and changes in the climate of the region. The land features of the area in the images of May 18 and 21, 2020 appear in dark brown to brown, representing that the land is wetter due to presence of a high amount of water content in soil compared to the images of subsequent dates, which exhibit the land in brown, light brown and very light brown signifying that the land got decreased in wetness due to increase of temperature and change in the climate. The FCC images show the change in the wetness of land in the study area.

## Did permafrost thaw cause the Norilsk Oil Spill?

**Figure 10:**
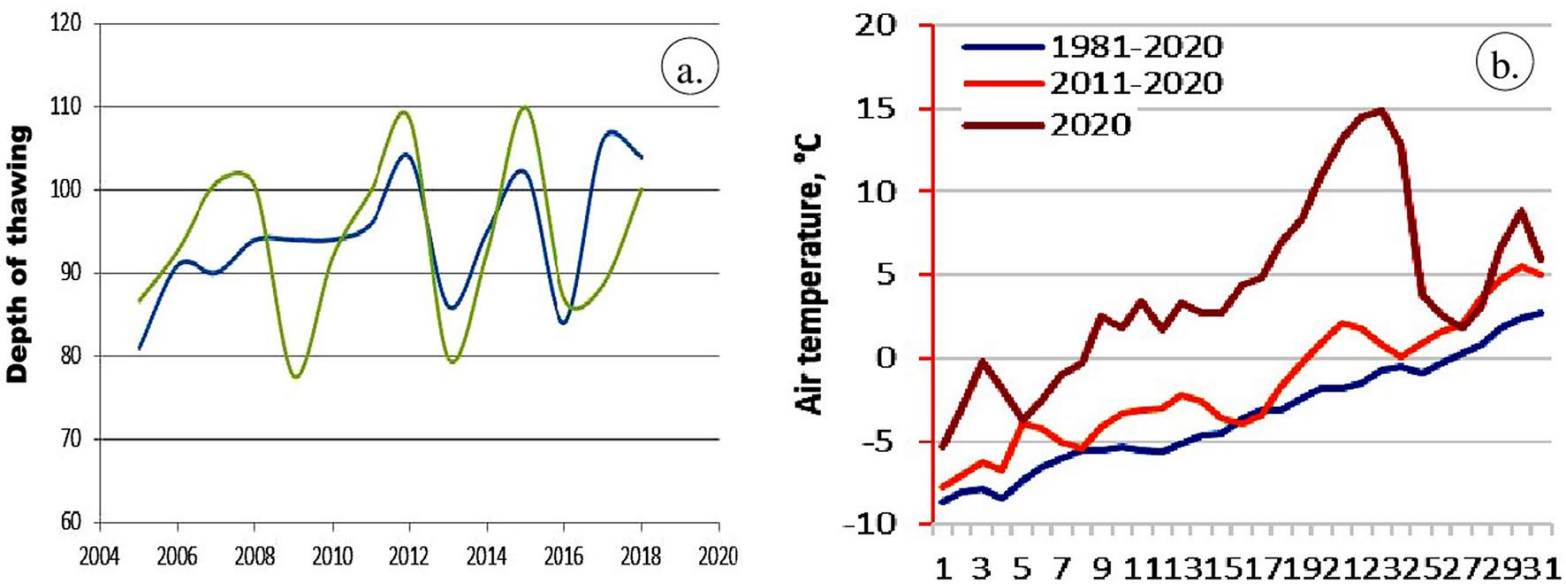
(**a**) Comparison of the calculated (green) and observed (blue) variations in the depth of seasonal thawing in Talnakh in the 2005–2019 period, (**b**) Mean daily air temperatures in May in Norilsk averaged over the periods 1981–2020, 2011–2020 and in 2020 [https://www2.gwu.edu/~calm/data/north.htm].

Official statement of Norilsk Nickel named the climate change and thawing permafrost as the main factors that caused sinking of supporting piles in the foundation of the storage tank^41,57^. We examined this hypothesis using the dynamical permafrost model^96^. We validated the model by comparing the calculated depth of seasonal thawing with the observations in Talnakh at R32 site (Fig. 1) of the Circumplar Active Layer Monitoring (CALM) project in the period 2005–2019 (https://www2.gwu.edu/~calm/data/north.htm). Figure 10a illustrates the high performance of the model, which replicated the observed interannual variations of the seasonal thaw depth with high accuracy.

We calculated changes in the depth of seasonal thawing over the 1980–2019 period. Although we did not find positive trends, results demonstrate significant interannual variations of the seasonal thaw depth in Norilsk. Such variations in the past two decades (Fig. 10a) may have caused uneven ground settlement around the structure, which accumulated over time and favoured water ponding and thermokarst. Combination of these factors, markedly warmer than normal weather in May 2020 (Fig. 10b), and intensive meltwater streams eroding the surface may have weakened the pile foundation underneath the structure and ultimately led to the collapse of the oil tank.

**Figure 11:**
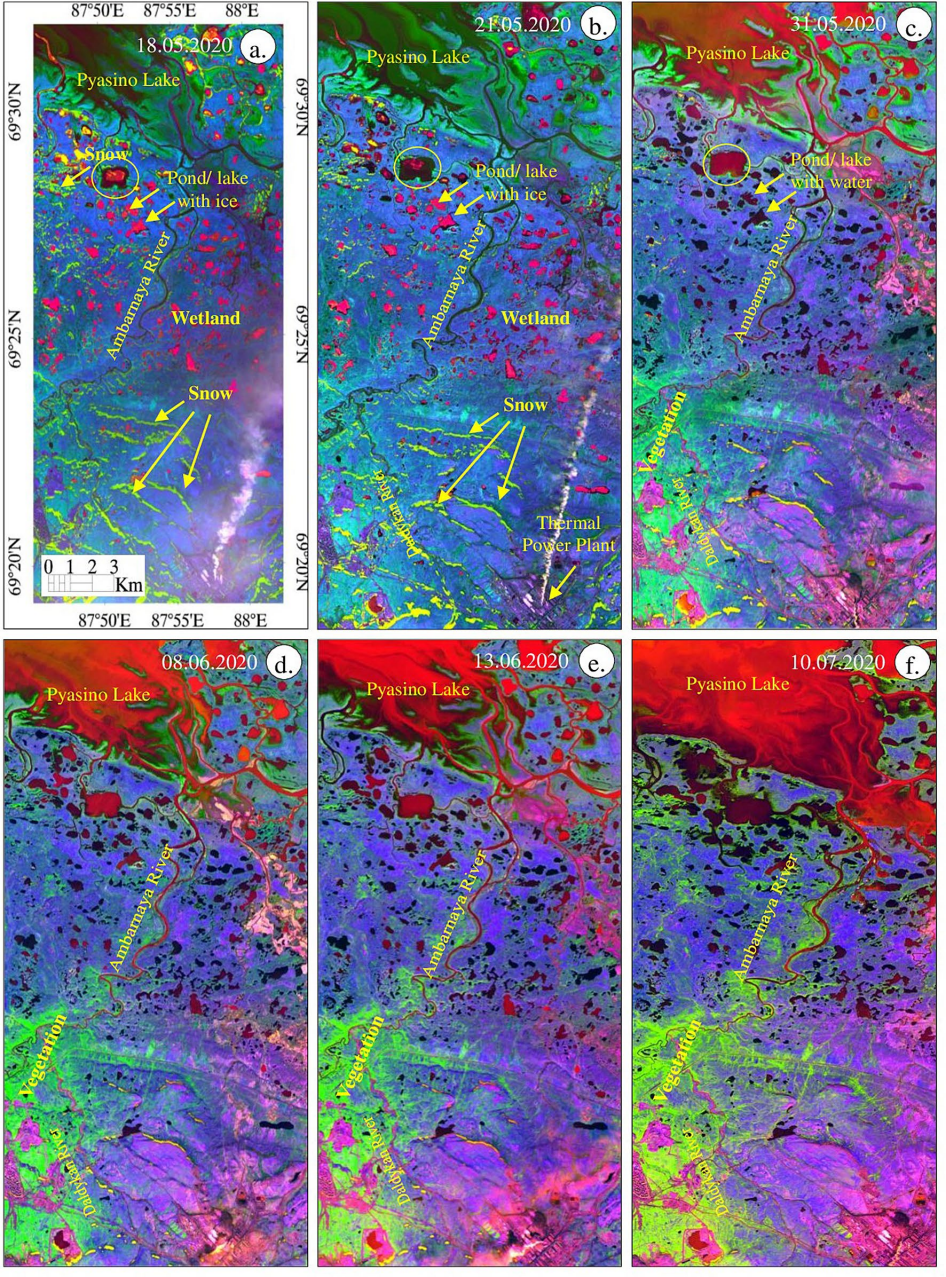
(**a**–**f**) Sentinel-2 decorrelated images of the spectral bands 3, 8, and 11 of the May 18 to July 10, 2020 [ENVI 5.5 https://www.harrisgeospatial.com; https://sentinel.esa.int/] showing the presence of snow, water, vegetation, and wetland features.

Our conceptual model based on the analysis of the meteorological data and permafrost modelling is consistent with the results based on the Sentinel-2 data. The SWI images evidenced the melting of snow and ice between May 21 and 31, 2020, which is further confirmed by the NDWI images demonstrating the occurrence of more water in the area from May 31, 2020 due to ice and snow melt. These findings are consistent with the NDVI and FCC images, which showed wetland with no or very poor vegetation till May 21, 2020, and an increase of vegetation thereafter. However, all the images could not well discriminate the snow, ice, water, vegetation, and wetland clearly, and therefore a decorrelated image was developed using the spectral bands 3, 8 and 11 of Sentinel-2 acquired between May 18 to July 10, 2020, since these bands have characteristics absorptions of oil, snow, ice, water, vegetation, and wetland (see Sect. "Conclusions"). The resulted images are given in Fig. 11.

The images of May 18 and 21, 2020, show the occurrence of snow in light green to yellow as warped patches with fine texture. The existence of ice in water bodies appears as pink dots in parts of the land (circled in Fig. 11). The Pyasino Lake exhibits dark green to light green with fine texture, possibly due to the presence of snow, ice and wet soil in the lake. The land appears in dark purple and has no or few vegetation. Both images show the fumes of thermal and power plants in shades of white and linear features. The images between May 31 and July 10, 2020 show the disappearance of the snow. The Pyasino Lake appeared in dark green to light green turned to green–red to red with fine texture which may be due to the expansion of condensed cold water to water in the lake (see FCC images in Fig. 9). The significant increase in the amount of water was observed from May 31, 2020. The Sentinel-2 images best showed the changes in the snow to water from May 18 to 31, 2020 (for example yellow circled) as well as the increase in water quantity in the Pyasino Lake. The image discriminated vegetation of the area in light green and the increase in the growth of vegetation is interpreted along the Daldykan and Ambarnaya rivers and around the Pyasino Lake from May 31, 2020. The wetland that appeared in dark purple between May 18 and 21, 2020 turned to light purple, possibly due to an increase in temperature in the region. All features of the area are well discriminated in different tones over the images and the MSI sensor of Sentinel-2 has proved its capability in discriminating the features and monitor their changes during the dates.

## Validation of Sentinel-2 mapping

**Figure 12:**
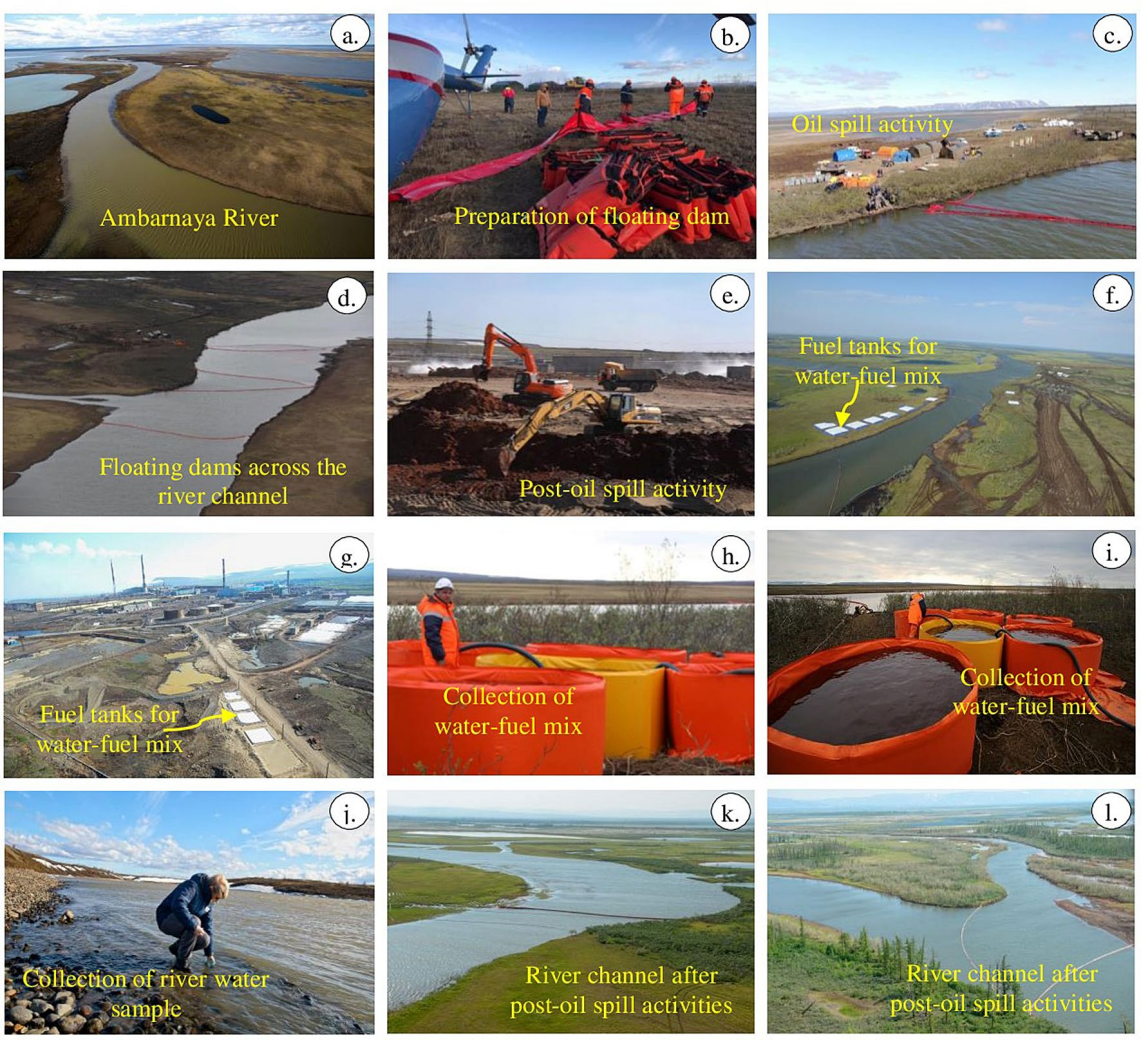
Aerial and field photographs showing (**a**) the oil spill in the Ambarnaya River, (**b**–**d**) the floating dam structures, (**e**–**g**) the measures taken for oil spill, (**h**, **i**) the skimmed off oil and the toxic mix of water from the river, (**j**) the sampling of water and (**k**, **l**) the present ecology condition of the area^41^.

**Figure 13:**
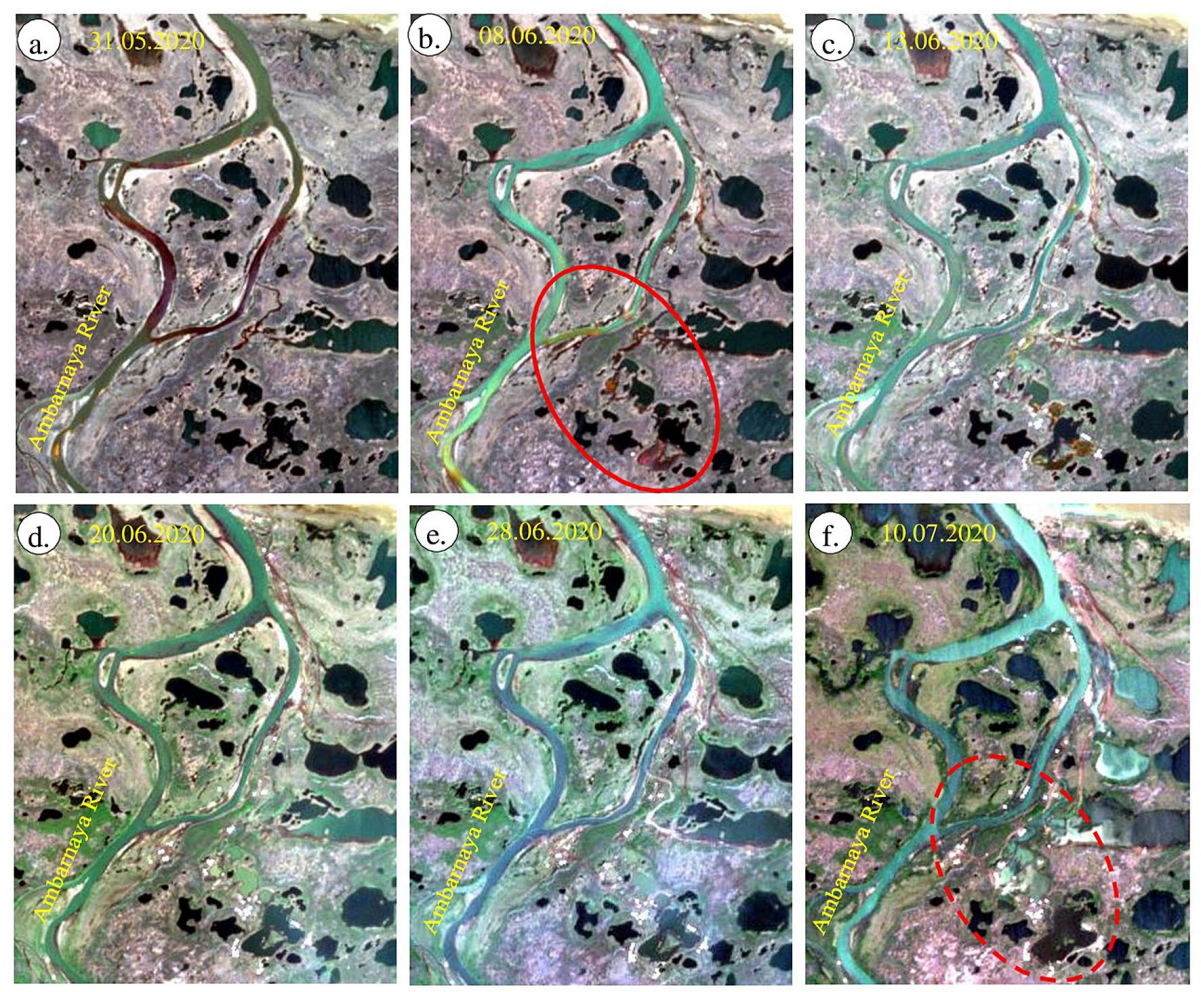
(**a**–**f**) True color images [R:4; G:3; B:2; ENVI 5.5 https://www.harrisgeospatial.com; https://sentinel.esa.int/] of Sentinel-2 from May 31 to July 10, 2020, show 1) the oil spill in the Ambarnaya River and the creeks and water bodies adjacent to the river channel in the crimson red [red elliptical, 31 May and 08 June, 2020], and 2) the area of contaminated soil removed, the special containers placed to store the skimmed off oil and the toxic mix of water collected from the river [red dashed elliptical as white spots].

In this study, the true-color images of Sentinel-2 dated May 31 and June 01, 2020, showed the occurrence of the oil spill in crimson red as a large patch over the Ambarnaya River (Figs. 5 and 12a)^41^. Monitoring of oil spill using the Sentinel-2 data available on June 06, 2020 acquired after six days from the date of the incident did not show a further spread of oil spill in the Ambarnaya River. It may be due to the activities of cleanup teams to stop the spread of fuel, further into the Ambarnaya River and prevent the oil spill movement towards the Pyasino Lake and Piyasino River which originates from the lake (Fig. 12b–d)^41,57–59^. The rates of the oil spill advance are not addressed in this study because of the restricted temporal resolution of the satellite data. Norilsk Nickel took great measures, and engaged clean-up teams, which placed floating dam and boom structures (Figs. 12b–d), removed tons of contaminated soil (Fig. 12e–g) and skimmed off oil and the toxic mix of water from the river using special containers (Fig. 12h, i)^41^. The company stated that they collected a total of 34,451 cubic meters of water and fuel mixture, and cleaned the land of 422,816 square meters (as of 28 September, 2020)^41,57^. It is believed that the rest of the spilled oil, if any, could have been removed in the subsequent dates, although it is difficult to remove them fully from the water and soil in the affected area. The post-oil spill activities improved the ecology condition of the area (Fig. 12j–l)^41^. The study of the stretch of Ambarnaya River using true-color composite images acquired during and after the oil spill showed the activities of the clean-up teams (Fig. 12). The MSI of Sentinel-2 has the potential to show the area before, during, and after the oil spill (Figs. 5, 6, 7, 8 and 9), and capable of monitoring the post-oil spill activities (Fig. 13).

## Conclusions

This study analyses the Sentinel-2 satellite imagery data acquired before, during, and after the oil spill from the fuel tank in Norilsk on May 29, 2020, and explores the hypothesis that changing climatic, permafrost and weather conditions may have caused the collapse of the structure. In summary, the Sentinel-2 data show the following: 1) disappearance of snow and ice in ten days between May 21 and 31, 2020 due to unusual warmth in May 31, 2020; 2) the change of water state in the Pyasino Lake between May 21 and 31, 2020 and the subsequent increase of the water level in the lake after May 31, 2020; 3) development of vegetation between May 21 and 31, 2020 and increase in vegetation greenness after May 31, 2020, and 4) the change of wetland to vegetated land between May 18 and July 10, 2020. All these changes are consistent with the conceptual model suggesting that abnormally high temperature in May 2020 may have contributed to the collapse of the fuel tank and oil spill in Norilsk. This study demonstrated one of the applications of Sentinel-2 data.
